# Supplementary material for: Complexome profiling of the Chlamydomonas psb28 mutant reveals TEF5 as an early PSII assembly factor
Source: Plant Cell. 2025 Mar 17;37(6):koaf055. doi: 10.1093/plcell/koaf055 (PMC12164590; doi:10.1093/plcell/koaf055)
Supplement: koaf055_Supplementary_Data [file koaf055_supplementary_data.zip › psb28_interactive.zip!/plots/intensity/qualityPlots/peptides/ATPVC_Cre06.g250250.t1.2.html]

### Cre06.g250250.t1.2

Trivial name: ATPVC  
SeqCoverage [median]: 23.60  
Mass: 41.96 kDa  
  
 

| Sequence | MissedCleavage | Mass [Da] | wtIntensity | muIntensity | Score |
| --- | --- | --- | --- | --- | --- |
| ASEYNNLK | 0 | 937.45 | 0.00 | 624.26 | 93.26 |
| EYHAPTEGSELTTAQAEQLKK | 1 | 2330.14 | 0.00 | 1775.99 | 85.51 |
| LEIPDLR | 0 | 854.49 | 4118.73 | 1075.81 | 72.14 |
| SQLNQITR | 0 | 958.52 | 3483.07 | 5343.53 | 124.74 |
| TSAAALGSNYK | 0 | 1081.54 | 360.56 | 1002.82 | 105.40 |
| TSAAALGSNYKLEIPDLR | 1 | 1918.02 | 0.00 | 1179.71 | 75.46 |
| TSTMMEAVVNK | 0 | 1209.57 | 311.56 | 838.10 | 112.75 |
| VEGMSTESYVQR | 0 | 1384.63 | 0.00 | 400.14 | 156.48 |
| VLDDFKAAAR | 1 | 1104.59 | 306.17 | 3954.51 | 68.66 |
|  |  |  | 8580.1 | 16194.9 |  |
